# Pseudomonas aeruginosa Endocarditis With a Large Mitral Valve Vegetation Causing Severe Mitral Valve Regurgitation and Cardiogenic Shock

**DOI:** 10.7759/cureus.61742

**Published:** 2024-06-05

**Authors:** Kriya Shah, Luis E Santiago, Tatiana Gusan, Tricia Gomez, Choudhry Poonam

**Affiliations:** 1 Medicine, Dr. Kiran C. Patel College Of Osteopathic Medicine, Nova Southeastern University, Fort Lauderdale, USA; 2 Internal Medicine, HCA Florida Northwest Hospital, Margate, USA; 3 Internal Medicine, HCA Florida Westside Hospital, Plantation, USA

**Keywords:** infective endocarditis, mitral valve replacement, severe mitral regurgitation, cardiogenic shock, pseudomonas aeruginosa endocarditis, pseudomonas infections

## Abstract

Infective endocarditis (IE) is characterized by the inflammation of the inner layer of the heart that can be caused by different pathogens. Pseudomonas aeruginosa is an uncommon source of IE. The clinical presentation is highly dependent on the patient’s medical history, societal factors, and valve involvement. This infection is associated with many unfavorable complications and high mortality rates. We present a case of P. aeruginosa endocarditis causing severe mitral valve regurgitation, leading to cardiogenic shock and an eventual replacement of the mitral valve. Prompt and sensitive antibiotics in combination with surgical consultation are vital to the survival of this condition.

## Introduction

Infective endocarditis (IE) is a life-threatening condition in which the lining of the heart, the endocardium, becomes inflamed due to microbial infection. Male sex, injection drug use, age greater than 60 years, prior IE, poor dental hygiene, indwelling intravascular devices, and cardiac implantable devices are risk factors for IE [1]. This condition can commonly manifest with symptoms of fever, chills, headache, malaise, weight loss, night sweats, and dyspnea. A thorough history and multi-system physical examination are essential to raise suspicion for this diagnosis. Auscultation is an important tool to use when diagnosing patients, as 85% of those with IE will present with a new or changed cardiac murmur [1]. A skin examination can yield important information with findings of Janeway lesions, Osler nodes, petechiae, and splinter hemorrhages. Patients may also have splenomegaly. Blood cultures and transthoracic echocardiograms aid in formulating the diagnosis of IE. The Duke Criteria is a useful tool to help determine the likelihood of this disease [1].

It is critical to diagnose and treat IE as soon as possible to decrease patient morbidity and mortality. Significant sequelae of IE can include heart failure, perivalvular abscess, pericarditis, stroke, meningitis, renal infarction, vertebral osteomyelitis, septic arthritis, lung abscesses, and pneumonia [2]. Treatment should be tailored to the responsible organism, although critically ill patients may warrant immediate empiric therapy after blood cultures are obtained. The most common organisms that cause native valve IE include Staphylococcus aureus (31% of cases), Viridans group streptococci (17%), and Enterococci (11%) [1].

Pseudomonas aeruginosa IE is a rare condition, comprising approximately 1.5% of all IE cases [3]. Earlier studies noted a higher incidence among intravenous drug users (IVDUs), predominantly affecting right-sided valves [4-6]. However, recent findings suggest a shift towards left-sided involvement, particularly in patients with prosthetic devices or abnormal cardiac anatomy, and non-IVDU cases [7].

Treatment presents challenges due to high rates of secondary complications, necessitating surgical intervention, and relapse. Recent series report relapse rates exceeding 33% despite appropriate medical therapy [4-6]. Common complications include embolization, intracardiac abscess formation, and heart failure, affecting over 50% of patients. Mortality rates have varied, with earlier series reporting rates exceeding 50%, which decreased with the introduction of higher doses of tobramycin in combination therapy [4-6].

Contemporary treatment typically involves at least six weeks of combination therapy with an antipseudomonal beta-lactam and high-dose aminoglycoside, with or without surgery [5-7]. Dual therapy is recommended to minimize resistance development and potentially enhance efficacy [5-6]. Various antibiotics, including antipseudomonal penicillins and carbapenems, have shown efficacy, while fluoroquinolone use is limited due to emerging resistance [6-8].

We present a case of Pseudomonas aeruginosa endocarditis with large mitral valve vegetation, causing severe mitral valve regurgitation and cardiogenic shock.

## Case presentation

A 57-year-old male with a past medical history of end-stage renal disease on hemodialysis, hypertension, third-degree atrioventricular block (AV) with a permanent leadless pacemaker (PPM), hypothyroidism, depression, and diabetes mellitus presented to the emergency department (ED) with complaints of fever and altered mental status. The patient presented with generalized weakness and confusion that worsened after his dialysis on the previous day. The fluctuating mental status had been present for three weeks with associated poor oral intake. He had a port infection six weeks prior for which he had received intravenous (IV) antibiotics for four weeks until the maturation of his left upper extremity fistula. 

On arrival at our institution, he was lethargic, confused, and oriented to person only. A cardiac and lung examination revealed crackles on the right lower lobe, decreased breath sounds, and a systolic murmur with an intensity of four out of six, best heard at the left fifth midclavicular line, with radiation to the left axilla. His vital signs were remarkable for a temperature of 37.9 °C, blood pressure of 85/55 mmHg, and a saturation of 92% on room air. Initial laboratory studies revealed a white blood cell count of 19.7 cells/UL (4,000-10,800 cell/UL), serum sodium of 127 mmol/L (136-145 mmol/L), potassium of 3.3 mmol/L (3.5-5.0 mmol/L), and serum hemoglobin of 8.3 g/dL (13.2-16.6 g/dL). Two sets of blood cultures drawn at the time of admission grew P. aeruginosa. IV fluids, cefepime, and vancomycin were administered at this time, and the patient was admitted for further management. 

**Figure 1:**
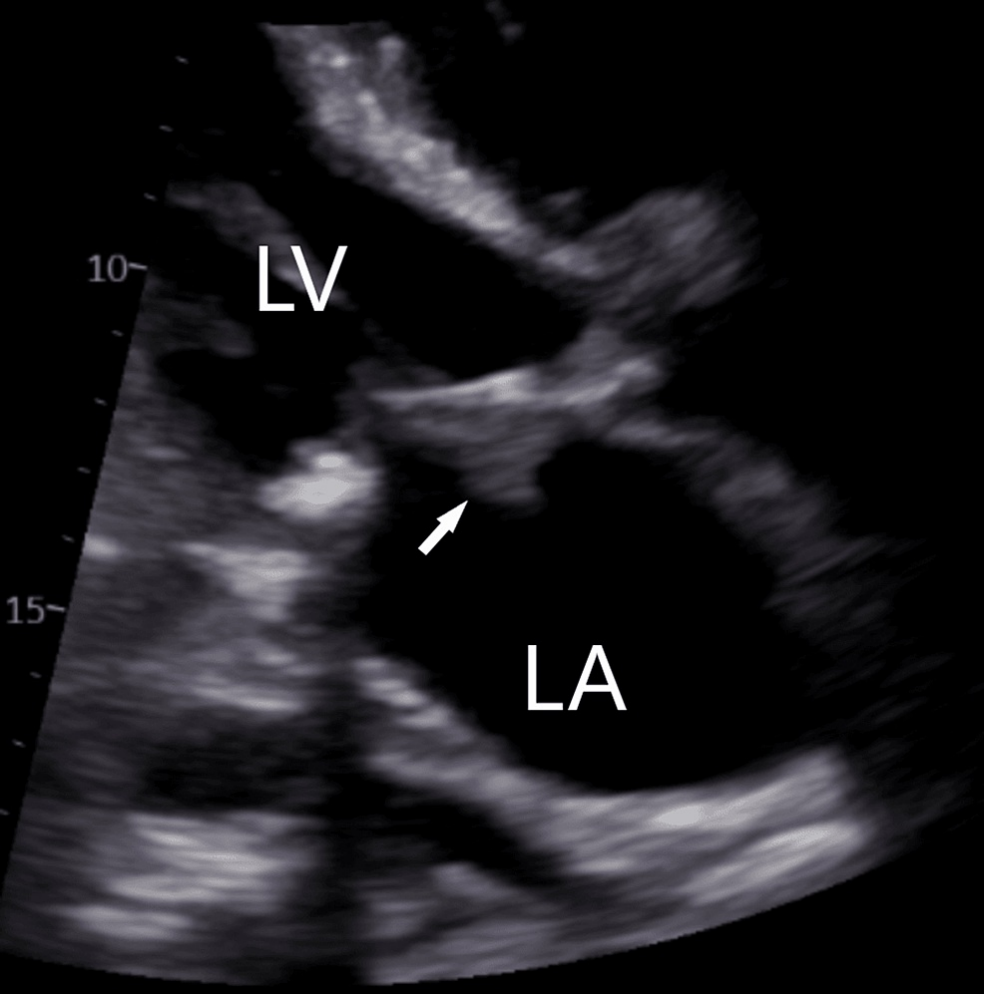
TTE showing an irregular and mobile vegetation measuring 17 mm x 14 mm on the body of the anterior leaflet (arrow). LA, left atrium; LV, left ventricle; TTE, transthoracic echocardiogram

**Figure 2:**
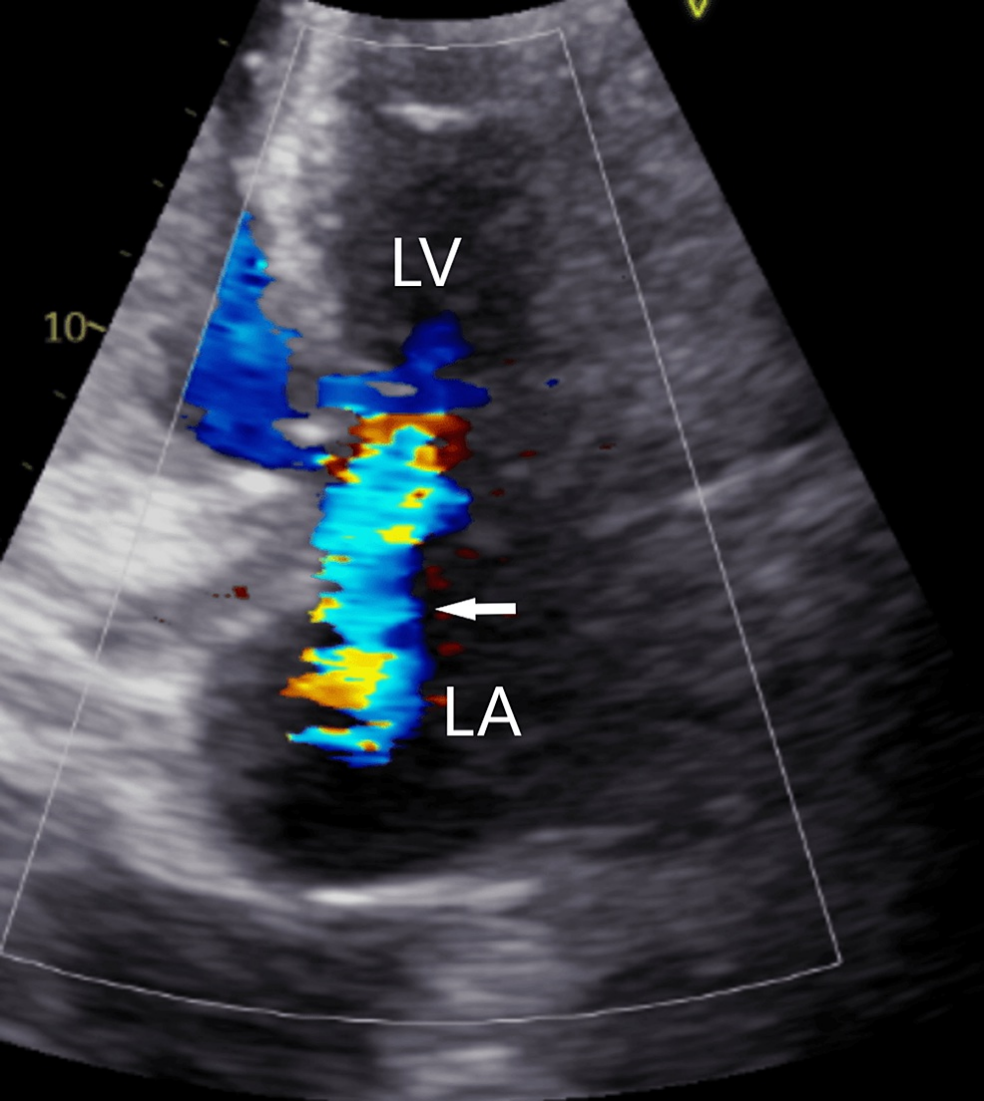
TTE showing moderate mitral regurgitation (arrow). LA, left atrium; LV, left ventricle; TTE, transthoracic echocardiogram

Initial chest X-ray revealed interstitial and airspace opacities throughout the thorax compatible with pulmonary edema. A moderate right pleural effusion and a small left pleural effusion were also present as well. A transthoracic echocardiogram was performed and revealed a left ventricular ejection fraction of 65%. On the mitral valve, there was a mild-to-moderate calcification of the posterior leaflet with irregular and mobile vegetation measuring 17 mm x 14 mm on the body of the anterior leaflet (Figures 1-2). Moderate mitral regurgitation was also present.

**Figure 3:**
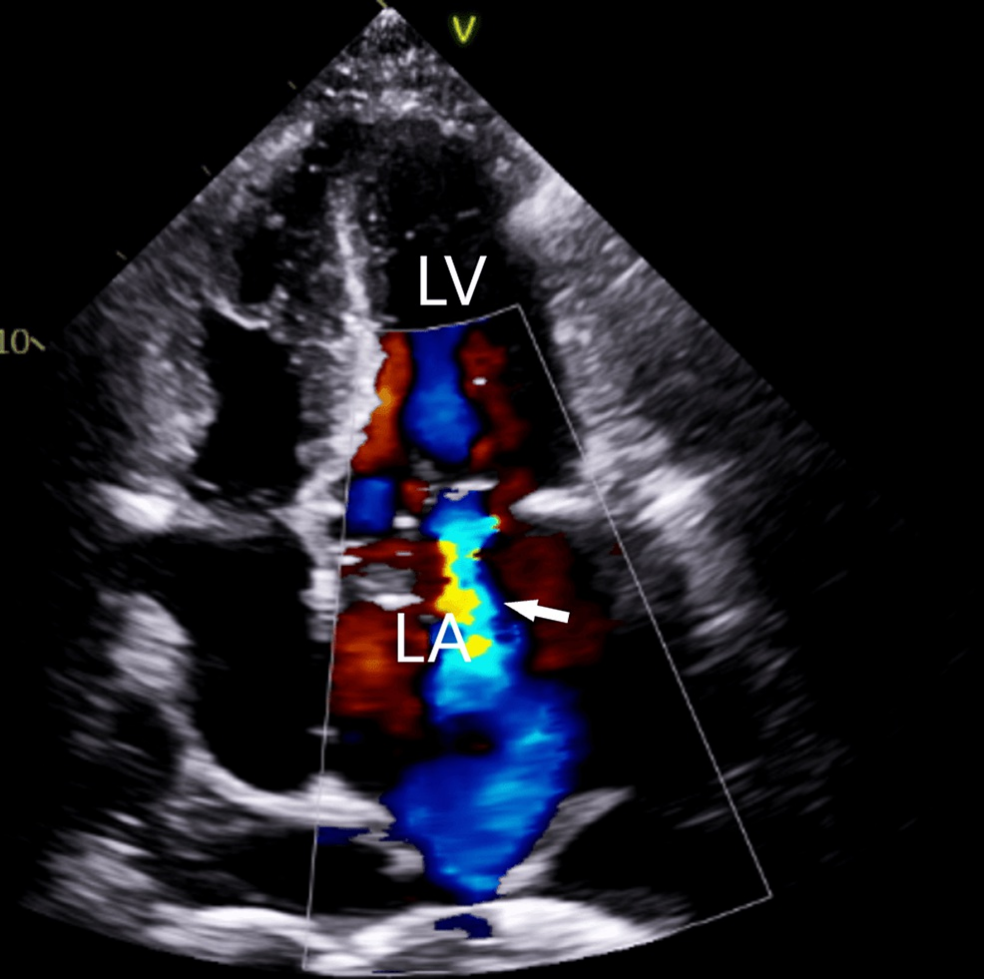
TTE showing severe mitral regurgitation (arrow). LA, left atrium; LV, left ventricle; TTE, transthoracic echocardiogram

**Figure 4:**
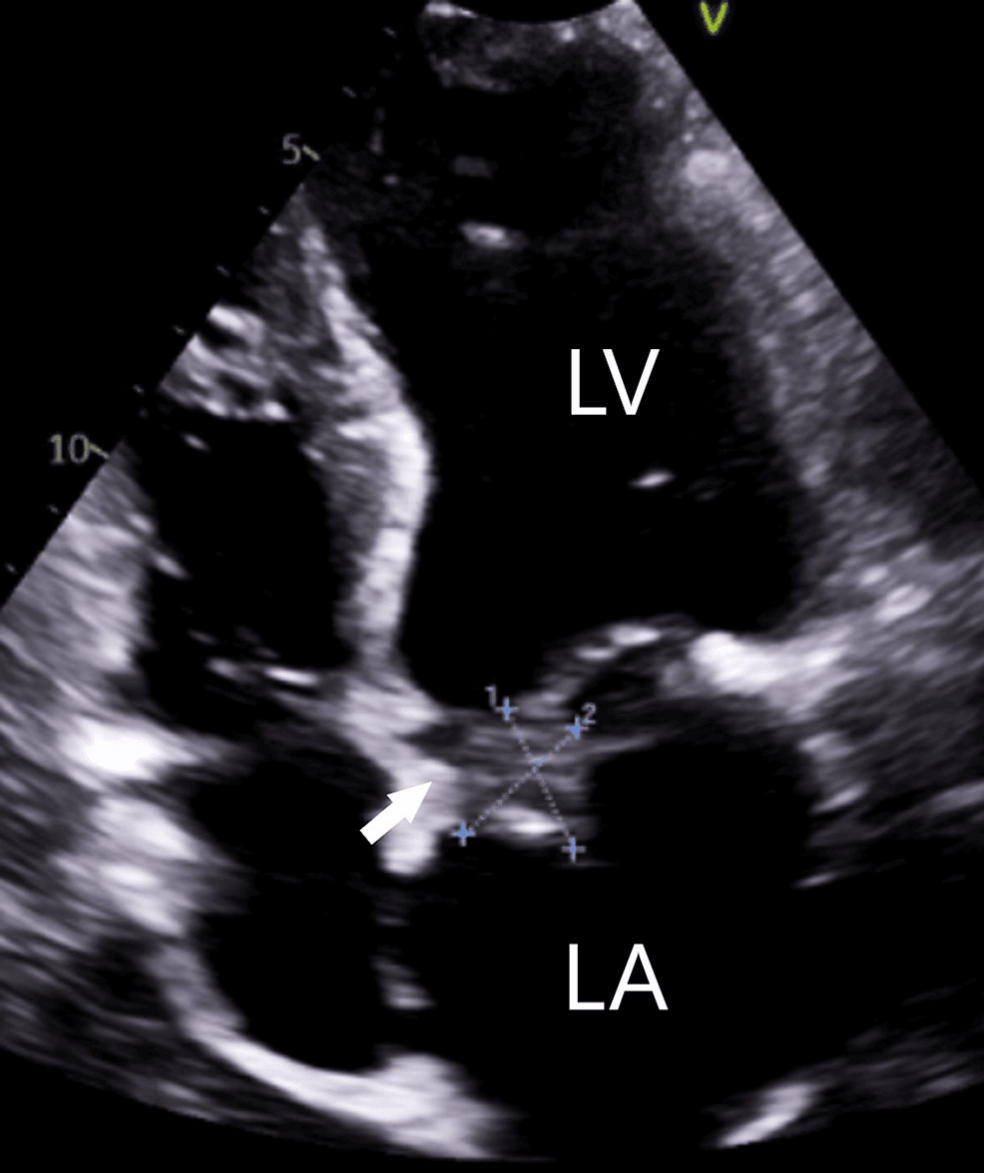
TTE showing a large 2.3 cm vegetation on the base of the posterior leaflet (arrow). LA, left atrium; LV, left ventricle; TTE, transthoracic echocardiogram

On day 4 of admission, despite the antibiotic regimen of IV meropenem and cefepime, a repeat set of blood cultures still revealed growth of P. aeruginosa. His condition progressed to acute respiratory failure and cardiogenic shock. He was started on vasopressors and inhaled nitric oxide for pulmonary hypertension, his pacemaker was removed as this was a possible nidus of infection, and a temporary pacemaker was placed. A repeat transthoracic echocardiogram revealed a moderate annular calcification of the mitral valve with a large 2.3 cm vegetation on the base of the posterior leaflet. The mitral regurgitation was more severe, and the tricuspid valve was also showing moderate regurgitation with the pulmonary artery peak pressure of 78 mmHg (17-25 mmHg) (Figures 3-4).

**Figure 5:**
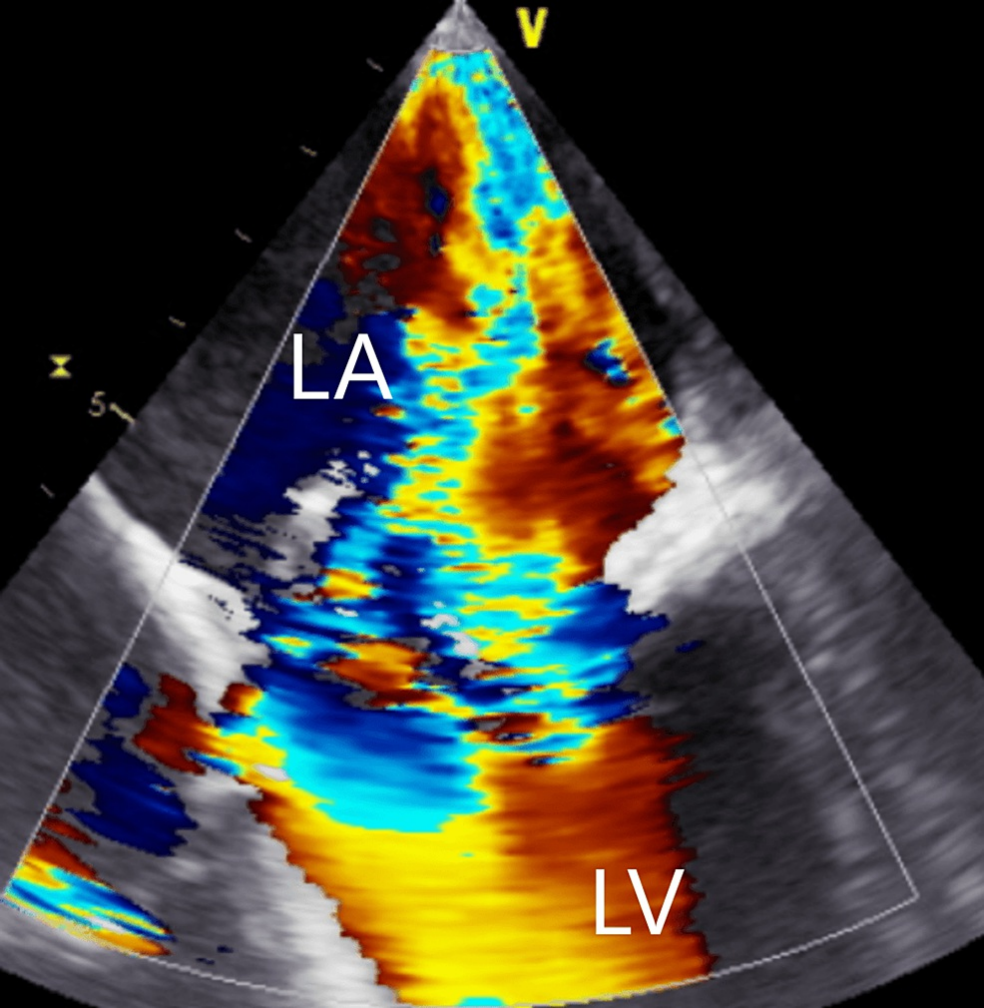
Intracardiac echocardiogram showing severe mitral regurgitation. LA, left atrium; LV, left ventricle

On day 6 of admission, the patient was evaluated by cardiothoracic surgery, and it was decided that emergent mitral valve surgery with Impella placement in the left ventricle would be performed. Intracardiac echocardiogram revealed wide open mitral regurgitation (Figure 5). Surgical intervention revealed perforation and destruction of anterior leaflets from large vegetation, severe mitral regurgitation with torn chordae, and severe pericarditis inflammation with pericardial adhesions. Lysis of pericardial adhesions was also performed.

After surgery, the patient continued on vasopressors, IV meropenem, and gentamicin. The patient’s hemodynamics improved as his pulmonary artery pressure and cardiac index started to normalize. Four days after surgery, the Impella device was explanted and the nitric oxide was stopped after his hemodynamics improved. His IV antibiotic regimen was continued for the next six weeks. Six days after surgery, the patient was extubated and gradually weaned off vasopressors. He made a successful recovery and was later transferred to the post-acute care unit. 

## Discussion

IE is created by an inciting injury and then followed by the presence of bacteria. The initial disturbance of the endocardium can be caused by a multitude of factors, such as turbulent blood flow, trauma from a foreign object such as a catheter or pacemaker, or recurrent use of intravenous drugs. This damage creates an environment for platelet aggregation and the coagulation cascade activation, which leads to the development of vegetation [9]. These vegetations are more common in high-turbulent areas of the heart, including the ventricular surface of the aortic valve and the atrial surface of the mitral valve [9]. The vegetations are then colonized by bacteria which originate from a distant site or hematogenous dissemination. 

IE is mainly caused by gram-positive streptococci, staphylococci, enterococci, and the HACEK organisms which include Haemophilus, Actinobacillus, Cardiobacterium, Eikenella, and Kingella, with a yearly occurrence of 3 to 10 cases per 100,000 people [9]. Lesser common organisms consist of Candida, gram-negative bacilli, and polymicrobial organisms [5]. This case specifically falls under the gram-negative bacilli, P. aeruginosa. P. aeruginosa endocarditis is considered very rare and accounts for only 3% of the total number of endocarditis cases [5]. There is a strong association between P. aeruginosa endocarditis and injection drug use, prosthetic heart valves, and pacemakers. Over 90% of P. aeruginosa endocarditis cases were in people who were IVDU [5]. Our patient, in addition to undergoing hemodialysis through his arteriovenous fistula, had a pacemaker for many months which was also a possible source of bacteremia. Clinically, patients present with pulmonary symptoms like cough and hemoptysis due to the high occurrence of the tricuspid valve involvement. These patients also have lung lesions that can lead to cavitations, as well as complications of annular abscesses [6].

IE has always been more prominent on the right side of the heart, but the clinical course of P. aeruginosa endocarditis has been changing in recent studies. A case series showed that P. aeruginosa IE has been associated with left-sided valve involvement, specifically the mitral valve. Of the 15 cases that were reported, 60% of them involved the left-sided valves and 53% had mitral valve involvement [10]. Our case similarly falls into this category as the patient had vegetation over the majority of the mitral valve. 

Duke Criteria are composed of pathological, major clinical, and minor clinical criteria. Pathological criteria consist of microorganisms in vegetation shown by blood cultures or pathological lesions confirmed with a histological examination. Major clinical criteria represent two separate blood cultures that are consistent with IE and an echocardiogram that is suggestive of IE (abscess, partial dehiscence of prosthetic valve, or new valvular regurgitation). Minor criteria consist of a predisposition, fever of >38.0 °C, vascular phenomenon (emboli, intracranial hemorrhage, conjunctival hemorrhage, Janeway lesions, purpura), immunologic phenomena (rheumatoid factor, Osler nodes, Roth spots), and microbiological evidence [11]. In our case, our patient had the presence of vegetation, new valvular regurgitation, and positive blood cultures.

Due to the severity of P. aeruginosa endocarditis, the treatment usually consists of antibiotics and surgical intervention. Regarding antibiotic regimen, IV dual therapy is recommended with one agent consisting of an aminoglycoside, and the second agent from another class of medications that is susceptible to Pseudomonas Aeruginosa for approximately six weeks [5]. It is also vital to get early surgical consultation based on the type of valve dysfunction and associated complications. Conditions that warrant early valve surgery include aortic or mitral valve regurgitation causing heart failure symptoms, development of an annular abscess or heart block, multi-drug-resistant pathogens, and continuing bacteremia for more than five days despite antibiotic therapy [12]. 

Endocarditis that is due to Pseudomonas is associated with poor health outcomes and an increase in fatalities. In a review of 15 cases of P. aeruginosa endocarditis, the mortality rate at the one-year mark was 26% [11]. Those who are above the age of 60 and have a prosthetic device have an even higher chance of mortality. Individuals who have received antibiotic and surgical therapy have improved prognosis than those who receive only antibiotics, especially in complex cases involving hemodynamic instability [5]. This was seen in our case as the patient was quickly hemodynamically decompensating and developed complications, which required an emergent mitral valve replacement.

## Conclusions

In conclusion, IE presents a serious threat to cardiac health. While most cases are caused by gram-positive bacteria, P. aeruginosa can lead to particularly severe infections, especially in individuals with prosthetic devices or a history of intravenous drug use. Diagnosis relies heavily on echocardiography and adherence to the Duke Criteria, with treatment emphasizing a combination of antibiotics and surgical intervention to address the infection's severity and complications. Given the significant mortality risk associated with P. aeruginosa endocarditis, especially in older individuals with prosthetic devices, timely and aggressive management is essential. Early surgical consultation is crucial for optimizing outcomes, particularly in cases with hemodynamic instability or complex complications. Overall, a comprehensive approach encompassing prompt diagnosis, effective antibiotic therapy, and judicious surgical intervention is essential in tackling P. aeruginosa endocarditis, aiming to mitigate mortality and improve patient prognosis.
